# Population dynamics and digitalization: implications for COVID-19 data sources in South Africa—a scoping review

**DOI:** 10.3389/fpubh.2024.1537057

**Published:** 2025-01-15

**Authors:** Monica Ewomazino Akokuwebe, Martin E. Palamuleni, Erhabor Sunday Idemudia

**Affiliations:** ^1^Faculty of Humanities, North-West University, Mafikeng, South Africa; ^2^Population Studies and Demography, Faulty of Humanities, North-West University, Mafikeng, South Africa

**Keywords:** Covid-19, digitalization, public health data, population dynamics, scoping review, South Africa

## Abstract

**Objectives:**

The study explores how humanity influences the development of digitalization on population dynamics during the changing times of the Covid-19 pandemic.

**Methods:**

A scoping review was conducted using PRISMA guidelines. Fifteen full-text articles were selected from 40 identified studies (2020–2023).

**Results:**

**T**hree themes emerged: (1) influence of humanity on the development of digitalization on population dynamics during changing times, (2) technical and ethical challenges, and (3) solutions to these challenges.

**Conclusion:**

Findings highlight the need for new opportunities for distinctive technical and ethical challenges in creating larger digital databases for population dynamics and how these databases may contribute to the advancement of population fields.

## Introduction

In recent decades, digital humanities (DH) and community practices have evolved, driven by technological advancements and sophisticated computational tools, leading to innovative DH techniques and frameworks. These developments disrupt conventional research methods and foster interdisciplinary collaboration across various professions, including social sciences. The data revolution has significantly impacted DH and population dynamics, enriching these fields through enhanced connections with traditional data sources (like censuses, administrative data, and surveys) and new big data sources, such as digital traces from internet and mobile phone use (1, 2). This shift has given rise to “digital demography,” which examines global population dynamics. The relevance of this evolution lies in its potential to address complex societal issues. By integrating diverse data sources and leveraging advanced digital tools, researchers can gain deeper insights into population trends, health disparities, and social determinants of health. This interdisciplinary approach enhances the ability to develop effective policies and interventions, ultimately contributing to the well-being of populations, especially in contexts like the COVID-19 pandemic (3, 4).

Population changes driven by birth, death, immigration, and emigration are crucial in the digital age and during transitions like the Covid-19 pandemic (2, 3). Despite its potential, current Covid-19 data for population research lacks methods to remove systemic biases and generalize findings (3, 4). The pandemic accelerated digital transformation, spurring AI development to learn about the virus and find vaccines. These efforts helped track the pandemic but also revealed gaps needing attention. Digital tools impact demographic processes as more people use them for communication, social interaction, and accessing essential services (4, 5). The relevance of these changes is significant. Understanding population dynamics in the digital age is essential for policymakers, healthcare providers, and researchers. Improved data collection and analysis can lead to better public health responses, more effective social interventions, and enhanced resilience in the face of future crises (5, 6). Digitalization and AI advancements also offer opportunities to address inequalities and improve access to essential services, highlighting the importance of bridging digital divides to ensure equitable outcomes for all populations (5, 7). The interdisciplinary nature of digital humanities and population science, particularly population dynamics, paves the way for new research on social determinants of health, creating new data sources for disease burden and pandemics.

Health equity, a crucial issue, especially in low-income countries (8, 9), is driving international and national debates. Integrating these fields helps understand disease epidemiology and generate data to address population health issues in South Africa (1, 6). This integration informs policies at local, provincial, and national levels, influencing health laws and identifying gaps. Strategic interventions and improved policies aim to advance population health, especially in grassroots communities. Learning from past data accomplishments in digital humanities (DH) and population dynamics reveals contemporary challenges in using statistical applications across these fields, including data theory and health epidemic data (3, 5). This has drawn scholarly attention to creating new data sources for COVID-19 population research. Key concerns in assessing quality data for such research in South Africa have been highlighted in several studies (1, 3). Additionally, the tools employed in DH during digitalization underscore the importance of social knowledge in developing new data sources for COVID-19 indicators. The advent of digitalization has revolutionized data collection, storage, and analysis in South Africa, particularly during and after the COVID-19 pandemic (1, 3). This shift has ushered in the era of “big data,” opening new avenues for studying population dynamics through digital data sources.

Key questions arise about how digital humanities should approach teaching population dynamics: Should the focus be on digitalization’s impact, the ethical and technological implications of using digital data, or resolving challenges in understanding its effects on human populations? Alternatively, should methodologies and practices in these fields take center stage? These issues are crucial for educators and non-educators alike. This study is important owing to the fact that population is dynamic and constantly changing in size and demographics, however, at the node of harnessing digital technological revolution of interdisciplinary fields of DH and population science applying the indicators of population dynamics. Significantly, it incorporates the systematic use of digital data sources of Covid-19 for research in population science and DH, as well as its implications for changing times in the era of digitalization trends. Therefore, using scoping review method, will identify knowledge gaps, explore setting research agendas, and provide a mapping of implications from emerging ethical challenges resulting from digitalization epoch of population dynamics during the changing times of Covid-19 pandemic. Hence, we will move to the method section that discussed in detail the scoping review analysis of existing literature.

## Methods

### Search strategy

This review was carried out in accordance with the Preferred Reporting Items for Systematic Review and Meta-Analysis (PRISMA) guideline. PRISMA is a set of requirements for systematic scoping, and meta-analysis reporting. Its focus is primarily on reporting reviews of randomized control trials; however, it is also used to write reviews of other types of interventions or further research conducted in social sciences and digital humanities. In this study, a scoping review was selected because the intention was to synthesize and map out emerging evidence on the influence of population dynamics involving digitalization and its implications for new data sources for population research in South Africa, in the context of an intensified setting such as Covid-19, which is relatively new (9, 10). The intention of a scoping review in this area is to provide an overview of existing literature, identify existing gaps for further research, and for the review’s findings to influence public policy and guide social research practices. Findings may also contribute to strengthening interdisciplinary research between population dynamics and digital humanities in the context of a pandemic. Also, this will inform the advancement of new data sources drawn from or characterized by population dynamics and digital humanities due to Covid-19. The literature search targeted peer-reviewed and published journal articles. We searched bibliographic databases and reference lists of relevant scoping reviews and other articles. Keywords used in the scoping review search of the literature included keywords drawn from the databases and reference collections, population dynamics during changing times, population dynamics in the digitalization era, new data sources of Covid-19 for population research and population dynamics plus digital humanities (11–13). Also included were synonyms and population dynamics headings of each of the databases Medline/PubMed and Scopus.

### Design and steps followed in the review process

We conducted a comprehensive search across various bibliographic databases to compile relevant literature on the topic of interest. This included African Journals Online (AJOL), PubMed/Medline, ScienceDirect, Google Scholar, Google browser, Firefox Mozilla, Cochrane Library, Scopus, Web of Science, Pro Quest, and Embase. These databases were accessible through the North-West University Library in South Africa. The search was categorized into three main strategies:

1. Online Research Databases: AJOL, PubMed/Medline, ScienceDirect, Cochrane Library, Scopus, and Web of Science.
2. General Internet Browsers: Google Scholar, Google browser, and Firefox Mozilla.
3. Hand Searches: Pro Quest and Embase.

Based on feasibility and the time required to screen each hit, and following the proposal by Hategeka et al. (14), we decided to screen only the first 100 hits sorted by relevance, as it was believed that further screening would not yield additional relevant articles. Additionally, four reviewers conducted hand searches of the reference lists of identified studies to ensure no relevant studies were omitted (15). The electronic databases and university library websites shared similar search terms and publication requirements. Boolean searches were conducted in each database and university website, utilizing specific MeSH terms. A summary of these terms is included in Table 1. We also searched reference lists of relevant scoping reviews and other articles (Table 1). Keywords used in the scoping review included those related to population dynamics during changing times, population dynamics in the digitalization era, new data sources of COVID-19 for population research, and population dynamics plus digital humanities (12, 13). Synonyms and population dynamics headings from each database, such as Medline/PubMed and Scopus, were also included. Table 1 below presents the inclusion and exclusion criteria for articles. The review process followed these steps:

1. The corresponding author developed the initial research question.
2. Relevant studies addressing the research question were identified using various search strategies and databases to avoid bias.
3. Studies meeting the inclusion and exclusion criteria were included in the analysis.
4. Data were charted using a narrative descriptive analytical framework.
5. Data were collated and summarized.

**Table 1:** Summary of search strategy.

**S/N**	**Databases**	**Search Terms**	**Filters Applied**
**A**	**Online databases for research**		
1	African Journals Online (AJOL)	(“Population Dynamics” [Mesh] OR “digital divide” [Mesh] OR “demographic trends” [tiab] OR “impact of digitalization” OR refer* [tiab] or digital health* [tiab]) **AND** (“Covid-19” [Mesh] OR “Covid-19 pandemic” [Mesh] OR “Covid-19 for population research”[Mesh] OR Covid-19 research[tiab] OR digitalization era[tiab]) **AND** (“Population research”[Mesh] OR Covid-19 and digital literacy[tiab] OR “Covid-19*”[tiab] OR “public health surveillance*”[tiab]) **AND** (“South Africa”[tiab] OR “Southern Africa”[tiab])	From 2020 – 2023 English
2	PubMed/Medline	(“Digital inequity”[Mesh] OR “social media”[Mesh] OR “demographic research for Covid-19”[tiab] OR “epidemiological monitoring for Covid-19 pandemic” OR refer*[tiab] or health digitalization*[tiab] **AND** (“Implications for Covid-19 data sources”[Mesh] OR “data sources for pandemic”[Mesh] OR “Covid-19 research”[Mesh] OR Covid-19 data sources[tiab] OR impact of digitalization[tiab]) **AND** (“Population dynamics during changing times”[Mesh] OR Covid-19 during changing times[tiab] OR “Covid-19 for public health*”[tiab] OR “digital inequity*”[tiab]) **AND** (“sub-Saharan Africa”[tiab] OR “South Africa”[tiab])	From 2020 – 2023 English
3	Science Direct	(“Covid-19 pandemic research”[Mesh] OR “monitoring and analysis of Covid-19 trends”[Mesh] OR “health data to inform public health decision and strategies”[tiab] OR “mobile health applications and telemedicine*”[tiab] OR refer*[tiab] or access to Covid-19 new data sources*[tiab]) **AND** (“Unequal impact of Covid-19”[Mesh] OR “dimensions of the COVID-19 pandemic”[Mesh] OR covid[tiab] OR coronavirus[tiab] OR “corona virus”[tiab]) **AND** (“Effects of Covid-19 pandemic and digital health”[Mesh] OR unequal impact of Covid-19[tiab] OR “health data of Covid-19*”[tiab] OR “Covid-19 and digital health*”[tiab]) **AND** (“Southern Africa”[tiab] OR “South Africa”[tiab])	From 2020 – 2023 English
4	Cochrane Library	(“Digital health technologies”[Mesh] OR “population dynamics and Covid-19”[Mesh] OR “mobile health for population research”[tiab] OR “Covid-19 and demographic dynamics*”[tiab] OR refer*[tiab] or access*[tiab]) **AND** (“SARS-CoV-2”[Mesh] OR “COVID-19”[Mesh] OR covid[tiab] OR coronavirus[tiab] OR “corona virus”[tiab]) **AND** (“Covid-19 trends”[Mesh] OR Covid19 and public health strategies[tiab] OR “Covid-19 new data sources*”[tiab] OR “Covid-19 for population research*”[tiab]) **AND** (“South Africa”[tiab] OR “southern Africa”[tiab])	From 2020 – 2023 English
5	Scopus	(“Data sources and population dynamics”[Mesh] OR “changing times and digital divide”[Mesh] OR “digital literacy and population research”[tiab] OR “digital health of population research*”[tiab] OR refer*[tiab] or access*[tiab]) **AND** (“Covid-19”[Mesh] OR “SARS-CoV-2”[Mesh] OR covid[tiab] OR coronavirus[tiab] OR “corona virus pandemic”[tiab]) **AND** (“Covid-19 data sources”[Mesh] OR digital divide[tiab] OR “ population studies and data sources*”[tiab] OR “Covid-19 pandemic and population research*”[tiab]) **AND** (“Southern Africa”[tiab] OR “South Africa”[tiab])	From 2020 – 2023 English
6	Web of Science	(“Covid-19 Health Services”[Mesh] OR “Covid-19 health care”[Mesh] OR “Covid-19 technological innovation”[tiab] OR “Covid-19 health services and new data sources*”[tiab] OR refer*[tiab] or access*[tiab]) **AND** (“Coronavirus”[Mesh] OR “COVID-19”[Mesh] OR covid[tiab] OR SARS-CoV-2[tiab] OR “corona virus”[tiab]) **AND** (“population dynamics”[Mesh] OR digitalization era[tiab] OR “digital health and population research*”[tiab] OR “demographic trends and Covid-19 data sources*”[tiab]) **AND** (“South Africa”[tiab] OR “southern Africa”[tiab] OR “developed countries”[tiab] OR “developing countries“[tiab])	From 2020 – 2023 English
**B**	**General internet browser**		
1	Google Scholar	(“Digital divide” [Mesh] OR “Population Dynamics” [Mesh] OR “demographic trends in changing times” [tiab] OR “impact of digitalization era” OR refer* [tiab] or digital health and covid-19 trends* [tiab]) **AND** (“Covid-19” [Mesh] OR “Covid-19 pandemic” [Mesh] OR “Covid-19 research data”[Mesh] OR Covid-19 pandemic and public health[tiab] OR Covid-19 in digitalization era[tiab]) **AND** (“Covid-19 research”[Mesh] OR Covid-19 digital literacy[tiab] OR “Covid-19 and population dynamics*”[tiab] OR “public health surveillance*”[tiab]) **AND** (“South Africa”[tiab] OR “Southern Africa”[tiab])	From 2020 – 2023 English
2	Google Browser	(“Health data quality”[Mesh] OR “Covid-19 data sources”[Mesh] OR “health and digital divide”[tiab] OR “population dynamics*”[tiab] OR refer*[tiab] or access*[tiab]) **AND** (“Covid-19 pandemic”[Mesh] OR “COVID-19”[Mesh] OR covid[tiab] OR coronavirus[tiab] OR “corona virus”[tiab]) **AND** (“Covid-19”[Mesh] OR Covid-19 trends[tiab] OR “implications of the Covid-19 pandemic*”[tiab] OR “health data sources*”[tiab]) **AND** (“South Africa”[tiab] OR “southern Africa”[tiab])	From 2020 – 2023 English
3	Firefox Mozilla	(“Health Services”[Mesh] OR “primary health care”[Mesh] OR “health care”[tiab] OR “health service*”[tiab] OR refer*[tiab] or access*[tiab]) **AND** (“SARS-CoV-2”[Mesh] OR “COVID-19”[Mesh] OR covid[tiab] OR coronavirus[tiab] OR “corona virus”[tiab]) **AND** (“Digital divide”[Mesh] OR population research[tiab] OR “population dynamics*”[tiab] OR “Covid-19 pandemic and data sources surveillance*”[tiab]) **AND** (“South Africa”[tiab] OR “southern Africa”[tiab])	From 2020 – 2023 English
**C**	**Hand searches**		
1	ProQuest	(“Mobile health applications”[Mesh] OR “population dynamics and digital health”[Mesh] OR “health information systems”[tiab] OR “digital transformation*”[tiab] OR refer*[tiab] or access*[tiab]) **AND** (“Coronavirus”[Mesh] OR “COVID-19”[Mesh] OR covid[tiab] OR SARS-CoV-2[tiab] OR “corona virus”[tiab]) **AND** (“unequal impact of Covid-19”[Mesh] OR population dynamics in changing times[tiab] OR “Covid-19 pandemic and population research*”[tiab] OR “public health surveillance and Covid-19 pandemic*”[tiab]) **AND** (“South Africa”[tiab] OR “southern Africa”[tiab])	From 2020 – 2023 English
2	Embase	(“Sources of Covid-19 data”[Mesh] OR “dimensions of Covid-19 research”[Mesh] OR “health disparities”[tiab] OR “digital health services*”[tiab] OR refer*[tiab] or access*[tiab]) **AND** (“SARS-CoV-2”[Mesh] OR “COVID-19”[Mesh] OR covid[tiab] OR coronavirus[tiab] OR “corona virus”[tiab]) **AND** (“pandemic and digital health”[Mesh] OR population dynamics and digital health[tiab] OR “Covid-19 impact on new sources of data*”[tiab] OR “strategic Covid-19 public health information*”[tiab]) **AND** (“South Africa”[tiab] OR “southern Africa”[tiab])	From 2020 – 2023 English

**Source**: Authors’ compilation, 2023; **Note that**: tiab = title or abstract; Mesh = Covid-19 Subject Heading; “*” indication truncation, searching for variation of suffixes and prefixes. TS = topic; CU = country/region. ti,ab. = title or abstract. The Boolean operators ‘AND’ and ‘OR’ were applied to combine searches and further refine the database search. The same terms were used for each database to ensure consistency in the search results. All searches were limited to publication dates between 2020 and 2022. English language was selected for the search.

### Inclusion criteria

The evidence for this scoping review was drawn from population studies involving population dynamics (birth, death, and migration) that center on new data sources in the digitalization era associated with digital-based humanities concerning the digitalization domains (16, 17). Population dynamics, defined as the study of how and why populations change in size and structure over time, has essential factors in population dynamics, including the rates of birth, death and migration (18, 19). Thus, population dynamics play a central role in many approaches to preserving biodiversity, which until now have been primarily focused on a single specific kind of approach. Population dynamics during changing times in the digitalization era were aligned with the definition in demographic research.

**Figure 1:**
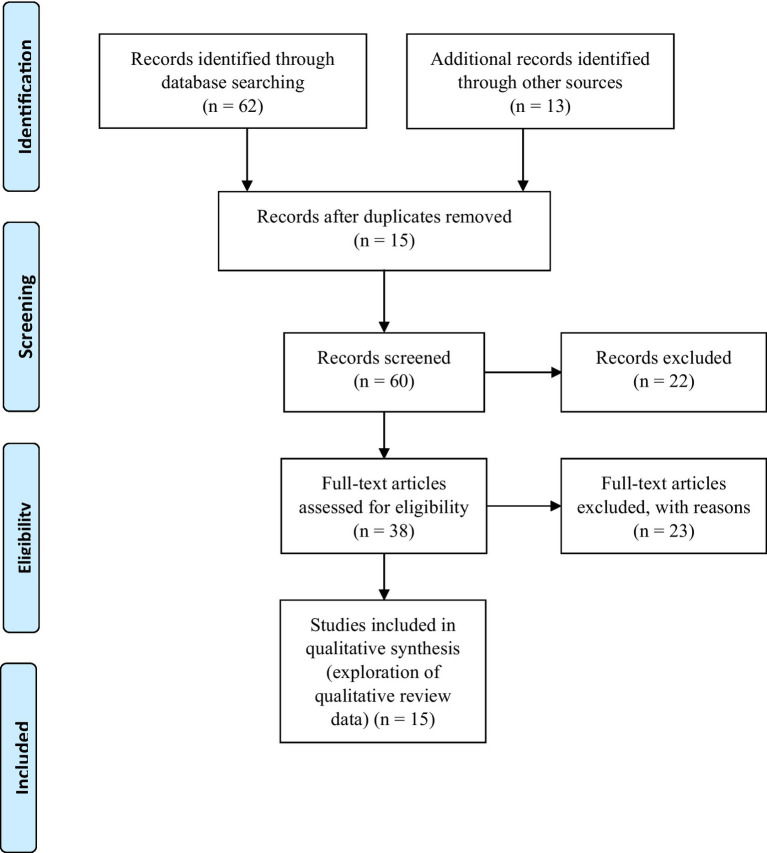
Flow diagram showing the selection and sample sizes generated from the selection process. Adapted from: Moher et al. (24).

Understanding demographic behaviors, especially in areas with gaps in traditional sources or measurements, can be challenging (20, 21). This review included quantitative and qualitative studies conducted in English, focusing on South Africa, Germany, Europe, and the United States between 2020 and 2023. These studies were published in peer-reviewed journals (Table 2). The review period from 2020 to 2023 aimed to assess the impacts of digitalization on population dynamics before and during the pandemic, highlighting efforts to enhance digitalization initiatives in the post-pandemic era. The search using various terms identified 75 articles: 62 from databases and 13 from other sources (Figure 1).

**Table 2:** Selected characteristics of the 15 articles, published between 2020 and 2023, included in the scoping review assessing the influence of humanity on digitalization development on population dynamics during the changing times of the Covid-19 pandemic.

+S/N	**Article & country**	**Title**	**Objectives**	**Methodology**	**Data sources**	**Findings**	**Limitations**
1	Mhlanga et al., 2020, South Africa (35)	COVID-19 and the Digital Transformation of Education: What Are We Learning on 4IR in South Africa?	The study sought to assess the influence of the COVID-19 pandemic in motivating digital transformation in the education sector in South Africa.	The study reviewed secondary data from peer-reviewed journals, reports, and newspaper articles, benefiting from recent publications on COVID-19 and the Fourth Industrial Revolution (4IR).	Secondary sources	Despite the widespread human suffering caused by the pandemic, it has provided an opportunity to evaluate the successes and failures of deployed technologies, their associated costs, and the potential for scaling these technologies to improve access.	However, its conceptual nature is limited by the evolving nature of 4IR and COVID-19.
2	Stats SA, 2020, South Africa (38)	COVID-19 Pandemic in South Africa – Demography Volume 1	The chapter examines women aged 15–49, focusing on the prevalence of non-communicable diseases (NCDs), causes of death, and associated risk factors, as well as the pattern of COVID-19 infections and deaths.	Statistics South Africa employed situational analysis to assess COVID-19's impact on employment, income, and health, using secondary data from 2020-2023, both quantitative and qualitative.	National surveys	The pandemic significantly impacts human populations and deaths, often neglecting population dynamics shaped by fertility trajectories. Historical events like wars, famines, and pandemics have led to short-term drops in fertility followed by later recovery.	The main limitation is the lack of fertility data during COVID-19, leading to conclusions based on literature review and existing data. The evolving pandemic impacts reporting, testing rates, and case definitions, potentially affecting estimates and factors beyond the researcher's control.
3	Kollamparambil et al., 2021, South Africa (37)	Behavioural response to the Covid-19 pandemic in South Africa	The objective of this study is to analyse the Covid-19 preventive behaviour and the socio-economic drivers behind the health-response behaviour.	The study employs bivariate statistics, concentration indices, and advanced multivariate techniques, such as probit models and specialized regressions, to address endogeneity and identify factors influencing response behavior.	South Africa’s National Income Dynamics Study (NIDS)—Coronavirus Rapid Mobile Survey (CRAM).	Behavioural responsiveness to Covid-19 was enhanced, and preventive behaviour is evolving over time; the use of face masks has overtaken hand washing as the most utilised preventive measure.	Pandemic literature, both pre- and post-Covid-19, is limited by reliance on cross-sectional analyses in developed countries, lacking multivariate approaches to distinguish mediating variables from true behavioral drivers.
4	Nwosu et al., 2021, South Africa (36)	Income-related health inequalities associated with the coronavirus pandemic in South Africa: A decomposition analysis	We hypothesize that the economic dislocation caused by the coronavirus will disproportionately affect the health of the poor.	The paper analyzed income-related health inequalities in South Africa pre- and during COVID-19 using NIDS (2017) and NIDS-CRAM (2020) data. Self-assessed health and household income were used to measure inequalities through concentration curves and indices, identifying key predictors of health disparities during the pandemic.	Fifth wave of the National Income Dynamics Study (NIDS) dataset conducted in 2017 and the first wave of the NIDS-Coronavirus Rapid Mobile Survey (NIDS-CRAM) dataset	The significance and magnitude of race, hunger, income and employment in determining socioeconomic inequalities in poor health, addressing racial disparities and hunger, income inequality and unemployment may likely mitigate income-related health inequalities in South Africa during the COVID-19 pandemic.	The study's main limitation is the data's nature. Pre-COVID-19 income and household size data are more objective than their COVID-19 era counterparts due to the lack of in-person surveys during lockdown. However, the sample's randomness mitigated potential bias, and analyzing the same individuals in both periods enhanced comparability.
5	Fischer et al., 2021, South Africa (34)	Changes in Perceptions and Use of Mobile Technology and Health Communication in South Africa During the COVID-19 Lockdown: Cross-sectional Survey Study	Increased technology use spanned various fields, but barriers like privacy concerns, unfamiliarity, and data costs were identified. The population had high COVID-19 knowledge, but reliance on web searches and social media over official sources raised the risk of health misinformation.	Between June 24 and August 24, 2020, South Africans were invited to participate in a survey through the Upinion mobile app, an online data collection tool. The survey gathered information on demographics, technology use during lockdown, and COVID-19 knowledge.	Survey through the Opinion mobile app, an online data collection resource.	Increased technology use spanned various fields, but barriers like privacy concerns, unfamiliarity, and data costs were identified. The population had high COVID-19 knowledge, but reliance on web searches and social media over official sources raised the risk of health misinformation.	The study may have selection bias due to device and data requirements for the online survey, which used a convenience sample. The survey, adapted from a prior study, was not validated or pilot-tested in South Africa, and self-reported technology use lacked independent verification.
6	Eike et al., 2022, South Africa (33)	How the COVID-19 Pandemic Alters the Landscapes of the HIV and Tuberculosis Epidemics in South Africa: A Case Study and Future Directions.	We contextualize the HIV/TB landscape in South Africa and analyze the direct and indirect impact of the COVID-19 pandemic on the country’s efforts to combat these ongoing epidemics.	This case study reviewed scientific literature, country reports, and news articles, sourcing data from platforms like PubMed, ScienceDirect, Google Scholar, and MedRxiv, as well as online national and agency-produced reports.	Secondary sources	The pandemic heightened social risk factors, increasing the spread of diseases. Individuals with HIV/TB face higher odds of severe COVID-19. It contextualized South Africa's HIV/TB landscape and analyzed COVID-19's direct and indirect impact on combating these epidemics.	This case study has limitations, including the dynamic nature of COVID-19 as of December 2021, which may affect its accuracy over time. It focuses solely on English-language sources, limiting generalizability and potentially introducing bias. Further research is needed to understand the interplay between COVID-19, HIV, and TB.
7	Zancajo et al., 2022, Europe (41)	Digitalization and beyond: the effects of Covid-19 on post-pandemic educational policy and delivery in Europe	This paper analyzes long-term responses articulated in the education sector; identifying three preponderant areas of response: the digitalization of the educational system, educational inequalities, and teachers’ development.	The paper reviews and examines long-term education policy responses from international and European documents, highlighting three key areas: digitalization, educational inequalities, and teacher development.	Secondary sources	Although the pandemic represents a common thread, countries have experienced the crisis differently according to the characteristics of their educational systems and the main problems the crisis has revealed.	Given the limited capacity of recovery plans to capture everything in terms of countries’ policy responses to the pandemic, future research should contemplate other data sources, including interviews with government and IO officials, among other key informants.
8	Alaimo et al., 2022, Europe (39)	The Medium-Term Impact of the COVID-19 Pandemic on Population Dynamics: The Case of Italy.	We compared demographic indicators from two periods (2002–2010: economic expansi- on and recovery, 2011–2019: recession and decline) with data from 2020 and 2021 to assess the impact of differing economic dynamics.	Use interpretative framework to analyze Italy's demographic balance, affected by COVID-19, comparing wide range of indicators (2002–2010 and 2011–2019) and (2020 and 2021), accounting for regional differences.	Interpretative framework using secondary sources	The COVID-19 pandemic has exerted considerable pressure on population dynamics, determining short-term (mortality increase), medium-term (more volatile migration flows) and long-term (fertility decline) effects.	The limitation of this study basically depends on the short time series representing demographic dynamics during the COVID-19 pandemic.
9	OECD, 2020, Europe (40)	Digital Transformation in the Age of COVID-19: Building Resilience and Bridging Divides, Digital Economy Outlook 2020 Supplement	Highlighting the growing importance and prioritization of digital technologies and strategies by governments, urging coordinated efforts for an inclusive digital transformation to build resilience and bridge digital divides post-COVID.	COVID-19 pandemic is accelerating both opportunities and challenges in digital transformation. Based on the OECD Digital Economy Outlook 2020, it outlines steps needed to build resilience and bridge digital divides in a post-COVID world.	Secondary sources	This emphasizes the growing role of digital technologies, with governments prioritizing digital strategies. It stresses the need for an inclusive digital transformation to build resilience and bridge digital divides as countries recover from COVID-19.	Limitations of this study include data limitations, potential selection bias due to the digital divide, generalizability issues, and the rapidly changing context or dynamic nature of the pandemic.
10	Barker et al., 2022, Germany (20)	A German Digital Grand Strategy Integrating Digital Technology, Economic Competitiveness, and National Security in Times of Geopolitical Change	This report systematically outlines the state of play in digital policy and Berlin’s current policy approach.	This report systematically outlines Berlin's digital policy and provides recommendations to enhance Germany's efforts in fostering a strong, high-performing European digital economy within an open, democratic, and rules-based framework.	Secondary sources	This scientifically outlines the provision of 48 recommendations for strengthening Germany’s efforts to build a confident, high-performing European digital economy embedded in an open, democratic, and rules-based digital order.	The limitation of this study could be the complexity of integrating diverse policy areas. Combining digital technology, economic competitiveness, and national security involves navigating various stakeholders and interests, complicating the implementation and effectiveness of the strategy.
11	Kalogiannidis et al., 2022, Germany (22)	The Impact of Digitalization in Supporting the Performance of Circular Economy: A Case Study of Greece.	The objective of this study was to investigate the effect that digitalization has on the performance of the circular economy.	The study used a quantitative cross-sectional survey design to assess variables such as digital practices for sustainability and digital business innovations (independent variables) and the performance of the circular economy (dependent variable).	Empirical analysis of quantitative data	There is a positive correlation between digital business practices and innovations and the success of circular economies, indicating that digitalization can drive the development of circular business models.	The challenges with the study design and execution are the primary factors contributing to the limits of this body of work. The COVID-19 pandemic was a serious limitation to all of the efforts that have been made.
12	Galanti et al., 2023, Germany (21)	Digital Transformation: Inevitable Change or Sizable Opportunity? The Strategic Role of HR Management in Industry 4.0	The study explores innovation processes in Italian factories, examining how digitalization can be an opportunity for creating a new way of working focused on adaptability, resilience, and openness to change.	This exploratory study used a triangulation of methods, combining in-depth interviews for qualitative data collection with both qualitative and quantitative analysis to ensure scientific rigor and preserve the strengths of each approach.	In-depth interviews of expertise	Future workplaces characterized by extreme versatility require workers to increasingly have both technical and soft skills as well as the ability to collaborate and build functional relationships.	The study's limitations include its exploratory nature due to limited research on Industry 4.0 in Italy, a skewed gender distribution with more male participants, and the non-generalizability of its qualitative approach to a larger population.
13	Shang et al., 2021, USA (25)	Effects of Pandemic Outbreak on Economies: Evidence from Business History Context	The coronavirus pandemic has highlighted the capitalist dysfunction showing that considering profit over people can be deadly.	The study analyzes the impact of pandemics on capitalist economies from a business history perspective, using both qualitative and quantitative methods to assess the economic effects on Coordinated Market Economies (CME) and Liberal Market Economies (LME).	Both qualitative and quantitative data	Online transactions and digital work platforms offer the opportunity to create a centralized database as an economic asset, essential for improving socio-economic conditions and mitigating the impact of the COVID-19 pandemic through digitalization.	The limitations of the study include its historical focus, limited scope of analysis, challenges in data availability, limited generalizability to modern contexts, methodological constraints in integrating qualitative and quantitative approaches, and the unique nature of the COVID-19 pandemic that may differ from previous outbreaks.
14	Sheng et al., 2021, USA (26)	COVID-19 Pandemic in the New Era of Big Data Analytics: Methodological Innovations and Future Research Directions	The paper introduces the literature review and analytics methods, discusses recent studies by management scholars, and concludes with methodological innovations for using big data to address global crises like COVID-19.	We present a review of the methodological innovations in studying big data analytics and how they can be better utilized to examine contemporary organizational issues.	Review	The paper explores methods in descriptive, diagnostic, predictive, and prescriptive analytics, and how they can be used to study 'black swan' events like the COVID-19 crisis and its implications for managers and policymakers.	The study's limitations are data quality and availability, technological constraints, the scope of big data, generalizability, ethical concerns, complexity of interpretation, and pandemic specificity.
15	Chan, 2022, USA (23)	Emergence of the ‘Digitalized Self’ in the Age of Digitalization	We examine whether there is such a new form of self as ‘Digitalized Self‘, with subsequent postulation, conceptualization and discussion.	This paper is reviewing the unprecedented and profound impact of digitalization upon our world and us in the Age of Digitalization, through interdisciplinary perspectives including psychology, neuroscience, psychiatry, sociology, anthropology, culture and history.	Review	Progressing from the Industrial Age to the Age of Digitalization when humankind has been undergoing changes in the process of digitalization, there is growing observation and evidence for the possible emergence of a new form of self, called ‘Digitalized Self’.	The study's limitations are a limited scope focused on specific cultural or demographic groups, data availability and privacy challenges, the evolving nature of digital platforms, potential subjectivity in qualitative methods, difficulty in maintaining methodological consistency across disciplines, and the digital divide affecting technology access.

Source: Authors’ compilation, 2023.

After removing duplicates, 38 potentially relevant articles for full-text review were retrieved, and 15 met our inclusion criteria (Figure 1). Articles were retrieved and screened by the corresponding author and three trained researchers. In addition, one author from our research team conducted multiple extensive electronic searches for all types of scholarly studies and gray literature (Table 2). The author extracted potential records using Google Scholar, African Journals Online (AJOL), PubMed/Medline, Science Direct, Cochrane Library, Web of Science, Scopus, Google browser, Embase, and ProQuest databases. Search terms were related to the influence of digitalization of population dynamics on humanity during changing times, exploring the technical and ethical challenges using population dynamics digital data sources, possible solutions to curb these challenges in understanding population dynamics and digital humanity, and location (using the term sub-Saharan Africa and the names of countries within that region).

The author examined reference lists of retrieved articles for additional studies missed initially and screened titles and abstracts to identify those needing further review. Both authors assessed the full texts of articles published in English between 2020 and 2023 (Table 2), focusing on qualitative research about the impact of humanity on the development of digitalization and population dynamics during the COVID-19 pandemic in sub-Saharan Africa (Table 2). Reviews and articles that did not meet the inclusion criteria were excluded. Initial findings indicated no strategy-based research in South Africa before 2020, apart from excluded topics like digital innovation and transformation driving new business models and markets (Table 2).

For this reason, most studies cited on population dynamics and digital humanities targeted the African context and perspective during precarious times. During the Covid-19 pandemic in 2020, there has been an increased domain of digitalization with its impact on demographic and digital humanities courses. Therefore, we chose the 2020–2023-time frame. Both authors independently examined the full texts of the remaining articles to verify eligibility for data extraction; after discussing any disagreements, we mutually confirmed that this final sample met all inclusion criteria and was appropriate for the thematic analysis. However, due to the small number of articles we found that addressed population dynamics in demographic and digital humanities techniques, we did not evaluate the quality of the included studies. To highlight the significance of the research to fill this gap, our objective for this scoping review was to identify any differences that will inform policy guidelines for improving the research in population dynamics in demography and digital humanities in South African educational institutions.

### Data extraction, synthesis, and analysis

Information such as authors, publication date, setting, data source, research design, the strategy implemented, and significant findings were extracted from each study using a data extraction form. The extracted information from the studies was both quantitative and qualitative and was analyzed and organized into thematic areas on strategies for digitalization implications in demographic research processes and digital tools. Identifying the quality and improvement in accessing the digitalization platforms in demographic and digital humanities research was significant, and many lessons should entail documentation in these perilous times. The primary outcome of interest was strategic so that humanity may influence the development of digitalization on the platforms of population dynamics during the changing times of the pandemic. Accessing effective strategies and approaches was accompanied by reported outcomes, which stemmed from quantified or self-reported improvement in the quality of demographic research and digital humanities. The indicators of population dynamics have indicated a deeper examination of the heterogeneity of these impacts, where it is necessary to understand who and under what conditions digital technologies can empower the quality of demographic research and digital humanities (22, 23).

The authors employed the use of Grading of Recommendations Assessment, Development and Evaluation (GRADE) system for grading the assessment of the quality of the evidence systematically collected from the bibliographic searches. Grading of Recommendations Assessment, Development and Evaluation (GRADE) is a tool for conducting evidence quality assessment and strength-of-evidence recommendation. This system was developed to provide transparency and to structure the evidence synthesis development process, such as through guidelines, systematic reviews, economic analysis, etc. (20, 23). According to the tool, the process of assessing the quality of evidence is unconnected from the process of formulating references, and in the case of reviews (either scoping or systematic), the quality of evidence reflecting the certainty of the effect estimates may be precise and accurate. Regarding the strength of the recommendation, the quality reveals the effect estimates to be adequate for supporting an explicit recommendation, thus helping the decision-making process (20, 21).

Thus, when using the GRADE strategy in a synthesis of the quality of evidence from empirical studies and qualitative analysis, the stages of evidence were categorized for each outcome of the studies on a four-level scale: very low, low, moderate, or high (20–24). In addition to understanding the social impacts of digital technologies, there is value in understanding the demographic characteristics of digital divides also from the perspective of using new streams of digital data for population generalizable measurement. This is an area where demographers have also begun to make contributions through exploring demographic dimensions of social media and internet use (25, 26).

## Results

### Analysis

#### Synthesis of results

This section synthesizes the data and findings from the scoping review, offering a comprehensive understanding of the study’s research themes. We extracted the following information from each study. We organized it in a Microsoft Excel database: title, authors, publication date, study location, study design, sample characteristics, assessment, theoretical framework, reproductive or maternal health program, data collection methods, intervention characteristics (i.e., number and types of intervention strategies, components, individual or couple-based approaches) and outcome measures. We also extracted findings from studies that specifically focused on the ‘new era of digitalization in population studies,’ ‘how demography has witnessed a data revolution,’ ‘digital transformation in the age of Covid-19,’ and ‘digital technologies for a new future.’ First, in the review modus operandi, we specified the number of subsection analyses to explore the aims of the quality of diversity and whether these were not comparable between studies, with the influence of contextual factors on population dynamics during changing times in the digitalization era and its implications for new data sources of Covid-19 for population research as a primary research focus. We performed a subgroup analysis focused on intervention strategies to show their effectiveness, or lack thereof, on the demographic outcomes interwoven in the digital humanities in the precarious times of Covid-19 and the digital era.

### Characteristics of the included studies

This section summarizes the key features of the studies reviewed in this research. This study showed the methodologies used in the 15 studies included 13 systematic review studies (qualitative; 86.7%) and two case presentations (13.3%). The systematic review studies consisted of one single-subject design (*n* = 1), while the case presentations consisted of two narrative literature-based studies conducted at a time. The first case cohort was centered on existing data sources for population dynamics that interest demographers, and the second focused on new sources of digital data hubs. Out of the 15 studies, six were both solely and partially conducted in South Africa (27–32), three in Europe (33–35), three in Germany (36–38), and three in the United States of America (United States) (39–41). According to the articles that exclusively focused on population dynamics in digital humanities, grouping studies corresponded to the interdisciplinary relationship of both fields (*n* = 13; Table 1) (29–41).

However, thematic analytical methods were employed. The thematic analysis of the 15 studies in this present study revealed that digital humanities could be considered the field where humanists meet with and use digital resources, methods, tools and technologies. The importance of digital humanities methods enables the mixed method of bringing together sizeable amounts of interdisciplinary research. They transform the idea of what humanity research can be, giving us new ways of seeing past and present ways of life. This further contributes to future research in demographic history. The data focused on looking at current research challenges in these fields and asking where digital humanities tools could be most beneficial (2, 26). Complex research topics such as long-term trends in population dynamics and demographic processes are central in many demographic studies. They would, of course, benefit from more use of automated records linkages. One of the main focuses on new data sources of Covid-19 for population research is to research the growth and structure of human populations on human stratification and social diversity on population health (19, 35). This is characterized by strong continuity in producing high-quality research on demography, population-based health and human development studies, and behavioral and social science approaches to the Covid-19 crisis. Most studies on population dynamics in the digital era need more data sources for Covid-19 in South Africa (30, 32). However, three themes emerged from the scoping review.

### Influence of digitalization of population dynamics on humanity during changing times

This section discusses how digitalization influences population dynamics on humanity during evolving times. This study revealed that four studies presented the influence of population dynamics on humanity during the pandemic: surge in the use of digital technologies (*n* = 4) (34, 36–38), social distancing (*n* = 2) (28, 38) and nationwide lockdowns (*n* = 3) (28, 33, 37). Two studies did not report taking any perspective on the discussion of interest (35, 39). All studies had a time-prospect viewpoint, and the periods varied from the start of the pandemic outbreak to the post-pandemic. Regarding the Covid-19 pandemic, a rise in the use of digital technologies is inherent in factors such as social distance rules and widespread lockdowns. Worldwide, individuals and organizations—including those in South Africa—have had to adapt to new modes of living and working. As a result, possible scenarios of the digital surge and research issues arose during the pandemic. Thus, increased digitalization makes firms and educational institutions shift to work-from-home (29, 42).

Four studies focused on the post-pandemic and the use of digital technologies to combat Covid-19 and its related health issues (13, 28, 31, 32). Thus, post-pandemic, digital technologies were seen to help in the fight against Covid-19, and the expansion of digital services helped to alleviate the impact of Covid-19 on the economy and boost the growth of South Africa. Various government and non-government agencies provided the public with authoritative, accurate, and accessible information, with strong measures via digital channels (13, 31). The information on the pandemic was provided on national portals, mobile apps, or social media platforms that covered outbreak statistics, travel restrictions, practical guidance on personal protection, and governmental responses. Reliable information from the government and other institutions helps people to make informed decisions about their daily routines and build public trust (30, 34).

Similarly, internet systems were developed to enable millions of students and employees to access their workplaces and places of education at home during the closures put in place to stop the spread of Covid-19. Teachers in South Africa have embraced online education and have streamed live lectures using Zoom, Microsoft Teams, and Google Hangouts (13, 29, 33). Businesses are using video conferencing and screen-sharing on electronic devices through digital platforms to bring their teams together in person worldwide, including in South Africa. In addition, digital data and artificial intelligence (AI) have been seen to help diagnose and monitor infectious viruses (28, 29). For instance, free Covid-19 analysis software was made available in South Korea by an artificial intelligence company for early viral detection and symptom assessment. Based on an examination of CT images, the software can identify, categorize, and create 3D models of lung damage brought on by Covid-19. The country also used contact tracing to combat the pandemic outbreak through mobile technologies such as GPS, cellphone masts and AI-powered big data analytics to help the government understand and manage the spread of Covid-19 within their communities (13, 29).

Among the 15 studies included, high-income countries, when compared with low-income nations, Covid-19 information was cost-saving among the general populace in five studies, cost-effective in two, and showed more effectiveness in one digital platform used in disseminating ample information on the spread of Covid-19, how to curb the virus, and uptake of vaccines. Also, some African governments used digital platforms in the sub-Saharan African regions to launch Covid-19 information services and debunk misinformation. For example, in South Africa, the national Health Department set up a WhatsApp service to provide information to locals, ranging from symptoms, prevention tips, and information on testing (28, 43). Notably, the service also dispelled false claims about cures, such as eating garlic and beetroot, to taking hot water baths, and it sensitized scammers looking to take advantage of people’s fears.

Eight studies performed thematic analytical explanations [20,22,23,24,25,27,2930]. In four of them, the findings were not altered (27, 31, 33, 37). Similar measures designed to reduce the risk of Covid-19 transmission through mobile money were adopted in Ghana, Nigeria and Uganda (43–45). In Togo, the government digitized social payments to transfer mobile cash to informal workers whose incomes were disrupted by Covid-19 (43). For instance, digital agriculture also paves the way for programs to lessen the impact of Covid-19 on food security. For instance, FarmIT offered agronomic assistance, market connections, and an e-commerce solution to farmers in Kenya (46, 47). Leading African platforms, like Mkulima Young and G-Soko, link farmers with suppliers and distributors, while the Zambia-based eMsika facilitates wholesale and retail commerce in agricultural goods (43, 44). Worldwide, in order to survive the Covid-19 storm, governments will increasingly rely on digital technologies. Since people may now access official information, enroll in online courses, apply for jobs online, send mobile money, and even receive telemedicine, regardless of where they live, digital platforms aid in the construction of more pandemic-resistant societies (43).

Furthermore, all studies reported using digital technology as an outcome, but none has detected the differences between the pandemic outbreak and the recent digital technology. The same was observed for five studies that evaluated population dynamics in digital fields (36, 38). Over the past two decades, these have transformed civilizations and advanced faster than any previous modernization, reaching almost 50% of the population in the developing world (28, 31, 33, 38, 41). Through enhancing connectivity, financial inclusion, trade access, and public service accessibility, technology can reduce inequality more successfully. For instance, frontier technologies that are AI-enabled are contributing to the healthcare industry’s efforts to improve life expectancy, and diagnose and treat diseases (27, 30). In addition, distance learning and virtual learning settings have allowed students who would otherwise be shut out of programs to participate. With the aid of AI, public services are also becoming less bureaucratically burdensome and more accountable through blockchain-powered systems. Moreover, big data can promote more accurate and responsive policies and programs (5, 48). To track and diagnose problems in agriculture, health, and the environment, as well as to carry out daily duties, digital technologies like data pooling and AI are employed today. Depending on the safeguards in place, data-powered technology can promote universal rights, empower individuals, and enhance well-being.

### Exploring the technical and ethical challenges using population dynamics digital data sources

This section examines the technical and ethical challenges of using digital data sources for population dynamics entails addressing concerns such as accuracy, privacy, and equitable access to data. This study revealed that only three studies reported the technical and ethical challenges using population dynamics within digital data sources (27, 34, 38). In one study, the emerging multidisciplinary approach of population dynamics and digital humanities has been supporting the initial research possibilities of applying digitalization methods and tools to study social phenomena (29, 36). Following a topical body of literature, this described platforms in which data have been misused, and how population scientists are being cautioned about the challenges hounding the subset of the methodological weaknesses encompassing population dynamics and digital humanities (5, 49). Five studies included the platforms that enhances the technical aspect of the underlying factors supporting population dynamics in the digital era (29, 32, 34, 36, 37), while three studies included the importance of digital platforms in the pandemic outbreak (30, 34, 36) and three studies included technologies that present fundamental biases in the digital data (33, 39, 41).

Digital platforms may be more pervasive than others, but not all individuals use them, and therefore are not captured by technologies that archive digital data; as such, this presents fundamental biases into the data, limiting assumptions that may be drawn from their outcomes (34, 38). However, the question of representativeness is not a new problem, nor it is unique to both population dynamics and digital humanities, yet bias may arise in standard survey procedures when put to use in digital methods that represent only non-institutionalized populations (12, 50). Although statistical problems are more complex for data sampling from digital platforms, even when grounded, true information may not exist, but this challenge is an opportunity to develop new sources of data for population research (5, 48).

Despite the potential of population dynamics in digital humanities, sound population studies using these data is still missing, including methodologies for addressing systematic biases and generalizing the findings to larger populations (2, 26). Also, migration studies are likely to be underestimated as a result of some migrant movements which are not documented or represented in publications indexed in bibliometric databases. This calls for future research integrating bibliometric data with complementary data sources to resolve some of the methodological issues. Despite these limitations, bibliometric data sources offer substantial benefits (26, 33) compared to traditional data sources like surveys.

Population dynamics study data can also be crowd-sourced and create platforms such as Geni.com and WikiTree that allows thousands of amateur genealogists to collaborate in building large-scale online genealogical databases such as the *Familinx* database, which includes 86 million individual records from around the globe, with data that go back as far as the 17th century (30, 34, 38, 41). This database was sourced from Geni.com, a collaborative social network that allows users to find and verify family relations. Online genealogies are a promising resource, as they cover long historical periods and are not restricted by national boundaries. However, on the downside, they are not representative samples and underrepresent low- and middle-income countries (LMIC). Despite their potential, sound population dynamics studies using these data is still missing, including methodologies for addressing systematic biases and generalizing the findings to larger populations (2, 26).

### Ethical dilemmas and challenges in digital data collection during COVID-19 in South Africa

The COVID-19 pandemic in South Africa has raised ethical concerns about digital data collection, including privacy violations, the digital divide, and data governance. Concerns arise from the security of personal information, unequal access to technology, and algorithmic bias and discrimination in using artificial intelligence (AI) for risk assessment and disease prediction (51). The COVID-19 pandemic in South Africa highlighted the need for transparency and accountability in using digital data for public health (52). Ethical dilemmas emerged, particularly regarding privacy and data ownership. The government’s use of mobile data for contact tracing raised concerns about potential misuse of personal information, highlighting the need for better transparency. Informed consent and data security were challenges in remote data collection methods, particularly in COVID-19-related online health surveys (53). The digital divide in South Africa highlighted the need for increased security measures, as cyberattacks posed a risk to sensitive health information, compromising the personal data of thousands of individuals (52, 53). The digital divide in South Africa has exacerbated data collection challenges (51, 53). Limited access to technology and poor internet connectivity in rural areas hinder inclusivity and result in underrepresentation. Biases in data collection methods can lead to skewed results, and digital tools may be more accessible to urban, tech-savvy populations (51, 52). The ethical use of data in public health initiatives has raised concerns about misuse, overreach, and privacy. The debate over AI and big data analytics for COVID-19 hotspot prediction underscores the need for strong ethical practices and transparent approaches to address ethical dilemmas and build public trust in digital health initiatives.

### Possible solutions to curb the challenges in understanding population dynamics and digital humanity

This section showed the platforms in addressing the challenges in understanding population dynamics and digital humanities which requires innovative technologies, accurate data, and interdisciplinary collaboration. Thus, this study revealed that emerging digital technologies offer researchers new avenues to collect real-time data. Nevertheless, the present ethical dimensions, considerations, and challenges are allied with conducting digital data collection in population dynamics research. Thus, the uncertainty has illustrated the crucial lines of research inquiry which warrant ethical consideration and increased ethical scrutiny, and restricting the conduct of research on population dynamics combined with digital humanities conceptual and operational frameworks raises ethical challenges. Nevertheless, the ethical merits of co-producing ethical practices of population dynamics and digital humanities create a mechanism to proceed with such research and, at the same time, address these technical and ethical concerns that center around uncertainty (54). The data revolution has already changed how we do demography, as evidenced by the digitization of historical censuses and population registers, and the creation of large-scale and open-access repositories of demographic and population dynamics data. The pace of these changes will likely increase as more researchers engage in ground-breaking research using digital data sources (50, 55, 56).

Regarding the issue of representativeness, prospective scientists have noted the difficulty in engaging in qualitative research using population dynamics and digital humanities combined methodology using digital data. Most of the digital data have been documented to avoid using several traditional qualitative methodologies that call for a social evaluation of each data component. Various populations have applied automated analytical strategies such as subject modeling to better understand the data content obtained from digital platforms (26, 56). Conversely, using digital data presents ethical challenges, and despite the easy access to digital data repositories, their use is only sometimes ethical. Indeed, most social media users value online privacy but usually do not suspect that their data will be used for research purposes, as the researchers may take steps to ensure the anonymity of users within their study. Precisely, these anonymous users are often easy to link by use of their fingerprint-like user metadata to specific individuals. Designating ethical standards for data uses protects users, especially vulnerable groups, from identification and possible discrimination, and privacy and protection in data use are extended beyond the individual (26, 50).

The availability of online data has also led population scientists to think long and hard about data security, privacy, and informed consent in the digital humanities era (26, 50). Ethical considerations must be a primary concern when designing demographic studies using digital or internet data. Social and population scientists, including sociologists and demographers, must adhere to ethical and transparent research practices, particularly as users’ privacy is constantly threatened in the online world (48, 57). Similarly, ethical management of population dynamics digital data is complicated by a varying landscape of data ownership. Many social media platforms share user data with approved third partners from domains including academia, law enforcement, and advertising. Recently, some new data sources for population dynamics have initiated and designed open algorithms to balance the need for individual privacy with the provision of scientific opportunity (33, 34).

Lastly, collecting, storing and managing digital databases and repositories can present formidable barriers for many population scientists and digital humanity researchers. In particular, using such data from research activities requires unique technical skills from both research fields. As stated before, representativeness and sampling pose challenges associated with digital data utilization, and these biases may limit the applicability of popular probabilistic statistical methods to these data (56). In contrast, some of these skills can be incorporated into existing teaching, often learned from inaccessible interdisciplinary studies. Practical steps in promoting population dynamics and digital humanities scientists may stimulate the consciousness and understanding of modern data science techniques for reproducible research. For instance, organizations such as the International Union for the Scientific Study of Population (IUSSP) offer training at their organized population conferences on the scientific panel ‘Big Data and Population Processes.’

Combining and complementing the proper use of digital data application in interdisciplinary research is another barrier in population and digital humanities research (26). Therefore, interdisciplinary studies should be supported with available resources to ensure researchers are adept at using new data sources of programming and computational methods with transparency to ensure reproducibility and dissemination of findings within an interdisciplinary setting (36, 48). A learning environment for population dynamics involving digital humanities research should be provided to foster collaboration and collective ability to promote innovative approaches to curb the methodological and ethical challenges facing the applicability of digital data involving population dynamics in the digital humanities research domain.

## Discussion

Findings of the scoping review revealed a scarcity of literature pertaining to the influence of Covid-19 on the population dynamics during changing times in the digitalization era, and new data sources of Covid-19 for population research in South Africa. There was an evident dearth of literature on this subject in the South African context, despite the high number of Covid-19 victims, exacerbated by strained healthcare resources and constrained access to full use of digital technologies to confront the pandemic (26, 57). Very few studies have considered alternative means and outcomes of the implications for new data sources for Covid-19. The evidence suggests that population dynamics can provide an alternative means to continue to note limited attention to population growth within the green economy and underscores the importance of increased attention to population dynamics, including the development of human-centered and rights-based policies.

There have been numerous reviews on the impact of the Covid-19 pandemic on topics related to digital health, including publication practices, public perception of science, and research funding overall (26, 41). The effect of the pandemic on primary data collection through digital-health technologies has had an incredible impact on subsequent data-sharing for secondary use, and its associated challenges for collaborative international scientific endeavors uncovered by the pandemic (57). The uptake of these data has been driven by timely access, standardization, and methodological developments. There is a need for renewed and deliberate efforts to integrate population studies comprising population dynamics and digital humanity fields into the academic curriculum for current and future population research. Present changing times in the digitalization era can enhance research skills through continued interdisciplinary or multidisciplinary academic collaborative research and professional development activities (1, 6). As previously mentioned, population dynamics are interwoven with digital humanities.

Digital humanities are a broad field of research and scholarly activity, covering the use of digital methods by the arts and humanities, which offer distinctive insights into significant social and cultural issues raised by the development of digital technologies (2, 42). Research in this field is methodological and interdisciplinary, involving multiple skills, disciplines, and areas of expertise with the investigation, analysis, synthesis, and presentation of data in electronic form (2, 38). Thus, understanding how population characteristics such as size, spatial distribution, age structure, or birth and death rates change over time can help scientists or governments make effective decisions. Some of the new data sources of Covid-19 for population research involve population dynamics during changing times (32). For instance, big data or digital trace data are widely used in deriving new understanding from massive aggregations of data, from text, images, and media, and analyzing it using computerized algorithms in creating advanced methodologies to resolve the gap between unexplained relations in micro- and macro-phenomena. Regarding the strengthening of population dynamics in research systems, other reviews found similar results and reported heterogeneity and scarcity of methodologically adequate studies.

In a scoping review, Alburez-Gutierrez et al. (56) and Planinc et al. (2) showed that there is an interplay between population dynamics and digital humanities that enhanced multidisciplinary research and has led to similar or improved population research outcomes, compared to research that is not focusing on population dynamics (34, 50). Therefore, a scoping review of the combination of social media platforms and traditional data sources will aid in boosting and enabling the further digital transformation of scientific research and incentivizing investment in new data sources in research and development. This may go a long way to support a robust response to and recovery from the Covid-19 pandemic (28, 37). The social media sources include web applications, smartphones, digital data, and online survey tools, while traditional data sources include such as questionnaires, census and survey data, and qualitative data (face-to-face). Thus, the data revolution and new data sources for population dynamics show how greater reliance on digital tools following Covid-19, ensuring trust, privacy, and data and consumer protection are needed in the digital environment (28).

Also, migration is central to population processes and represents an increasingly important component of population dynamics (28, 37). However, despite its growing importance, migration data remain expensive, difficult to collect, and burdened by inconsistent definitions used by different countries’ organizations. On the other hand, the spread of the internet and online social networks may provide unprecedented opportunities for studying global population dynamics, offering new data sources of Covid-19 for studying how migration impacts the pandemic in changing times in South Africa (27, 30). The harmonization of digital humanities and population science research will facilitate a conversation about improving migration data collection and developing new modeling approaches by bringing together the dynamics in migration data by employing interdisciplinary approaches and strategies for inferring information on migration from new forms of digital data and the modeling approaches for integrating different data sources (32, 38). The shift to digital has provided population scientists with numerous opportunities to pose insightful queries about the gathered data. With regard to population research, these questions focus on data accessibility and research applicability, addressing social issues such as data-driven healthcare and false and misleading information about COVID-19 (48, 49). A limitation of the review is the exclusion of studies published in other languages, which may have accounted for the absence of articles from Asia and central Africa. Other limitations are the exclusion of articles published before 2020, gray literature, and published reports which may contain helpful information on the impact of population dynamics during changing times in the digitalization era and its implications for new data sources of Covid-19 for population research in South Africa. Given these limitations, this review’s result, recommendations, and conclusions are limited to the covered scope. We recommend future reviews involving meta-analysis and those that will include or focus only on qualitative studies.

### Policy implications for research and practice

In the Covid-19 pandemic, we are confronted with a revolution in human collective intelligence. The vital descriptions of this revolution are concerned in “digital” replacement for “analogue,” design thinking, western culture and liberalism, human perception, structure and agency emerging as the main drivers of global change. This leads into a wider conversation about the expansion of a new discourse—the Digital Demographic Humanities, which aims to initiate a paradigm shift to develop comprehensive identifications of the link amid population dynamics and move away from the existing chauvinistic styles which prioritizes theory and data from the multidisciplinary nature of humanities “big data” relating population dynamics. Consequently, digital demographic humanities can be seen as scholarly response to these questions. This also indicates a step change over in digital humanities research. Thus, digital demographic humanities can mapped out vast literature on population dynamics that generates a niche of an improved knowledge of collaborative developments in digital humanity field. This study findings will raise the profile summary of scholarly social scientists in the areas of digital demographers in Humanity field, who are alien with it in the predicting future.

Also, as countries work to respond to and recover from the Covid-19 crisis, now is the moment to ensure an inclusive digital transformation with coordinated and comprehensive strategies that build resilience and digital bridges for a post-Covid-19 era. Sensitizing the public and creating awareness about the digital humanity field should be welcomed in higher educational institutions as it will attract research funding. Also, educational stakeholders should put more effort into policies that will transform and encourage multidisciplinary research collaboration that cuts across digital humanities and population science involving population dynamics. This approach will institute and build better strategies for new data sources of Covid-19 for population research in South Africa.

## Conclusion

The findings of this scoping review suggest the need for implementation research to determine how to scale up practical and new opportunities for distinctive technical and ethical challenges in creating more extensive digital databases for improving population dynamics research with digital humanities, and how these databases may contribute to the advancement of population research fields. Regardless of how the pandemic and its aftermath unfold, there is no doubt that digital technologies will continue to transform population research during changing times. Furthermore, the emergence of digital data and tools will further fuel the production of data, adding urgency to ongoing discussions centered around digital humanities in order to increase resilience against being unprepared for a future pandemic resulting in health crises and, in doing so, boost the significance of data flows between population studies and digital humanities. Thus, digital technologies can improve health outcomes by addressing health disparities. Responsible data use requires ethical data use, interdisciplinary collaboration, and new digital tools. Hence, understanding the long-term impacts of digital transformation on population health is crucial for future strategies and interventions.
